# Evaluating the design and implementation of the whole systems integrated care programme in North West London: why commissioning proved (again) to be the weakest link

**DOI:** 10.1186/s12913-019-4013-5

**Published:** 2019-04-15

**Authors:** Judith Smith, Gerald Wistow, Holly Holder, Matthew Gaskins

**Affiliations:** 1 Health Services Management Centre, University of Birmingham, Park House, 40 Edgbaston Park Road, Birmingham, B15 2RT UK; 2 Personal Social Services Unit, London School of Economics, Houghton Street, London, WC2A 2AE UK; 3 Centre for Ageing Better, Level 3, Angel Building, 407 St John Street, London, EC1V 4AD UK; 4 Charité – Universitätsmedizin Berlin, corporate member of Freie Universität Berlin, Humboldt-Universität zu Berlin, and Berlin Institute of Health; Division of Evidence-Based Medicine (dEBM), Charitéplatz 1, 10117, Berlin, Germany

**Keywords:** National Health Service, Purchasing, Planning, Commissioning, Coordinated care, Integrated care, Health and social care integration, Large-scale change, Commissioning cycle

## Abstract

**Background:**

Commissioning is a term used in the English National Health Service (NHS) to refer to what most health systems call health planning or strategic purchasing. Drawing on research from a recent in-depth mixed methods study of a major integrated care initiative in North West London, we examine the role of commissioning in attempts to secure large-scale change within and between health and social care services to support the delivery of integrated care for people living with complex long-term conditions.

**Methods:**

We analysed data collected in semi-structured interviews, surveys, workshops and non-participant observations using a thematic framework derived both deductively from the literature on commissioning and integrated care, as well as inductively from our coding and analysis of interview data.

**Results:**

Our findings indicate that commissioning has significant limitations in enabling large-scale change in health services, particularly in engaging providers, supporting implementation, and attending to both its transactional and relational dimensions.

**Conclusions:**

Our study highlights the consequences of giving insufficient attention to implementation, and especially the need for commissioners to enable, support and performance manage the delivery of procured services, while working closely with providers at all times. We propose a revised version of Øvretveit’s cycle of commissioning that gives greater emphasis to embedding effective implementation processes within models of commissioning large-scale change.

**Electronic supplementary material:**

The online version of this article (10.1186/s12913-019-4013-5) contains supplementary material, which is available to authorized users.

## Background

**Fig. 1:**
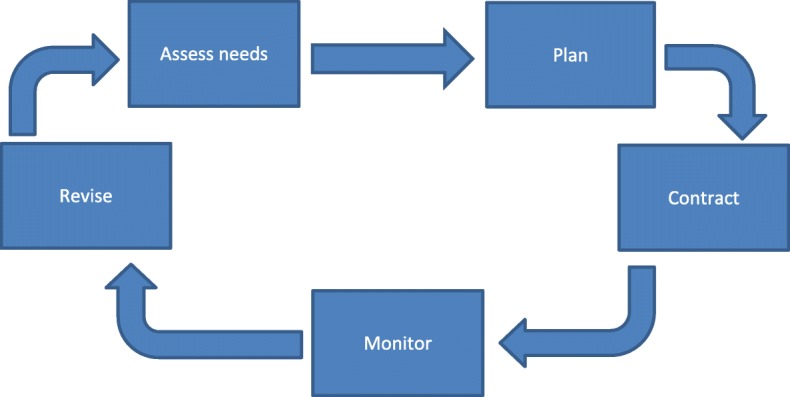
The commissioning cycle (Department of Health, 2003, after Øvretveit, 1995)

‘Commissioning’ is a term used in the English National Health Service (NHS) to refer to what most health systems call health planning or strategic purchasing. The NHS’ use of commissioning as distinct from contracting or purchasing has its roots in Øvretveit’s [1, 2] work on promoting health gain. His framework includes a strategic intent to improve health through needs assessment, planning, contracting, monitoring and review (see Fig. 1). Effective commissioning is regarded by NHS policy makers as crucial to achieving care that is responsive to patients’ needs and ensures value for money [3].

The need to join up commissioning across whole systems of health and social care services has been a particular feature of the debate about the contribution of commissioning to integration. In principle, it can provide a framework, first, for aligning strategic planning and investment decisions across NHS organisations and with local authorities to secure a balance and mix of services, consistent with local needs, resources and desired outcomes. Second, such frameworks can also be used to specify improved operational practices including the timely integration of different service components around the needs of individuals, service users and their families. From the perspective of integration, NHS and joint commissioning have a potentially substantial role in securing major changes to investment and working practices within health service and other organisations, as well as at the boundaries between them.

This paper aims to examine the use and potential of commissioning as a tool for large-scale change to plan and implement better integrated health and care services. To do so, we review evidence from an in-depth mixed methods study of NWL using a thematic framework derived both deductively from a review of literature on commissioning and inductively from our coding and analysis of interview data.

Given the degree of strategic and operational change implied by the scale of NWL’s goals for integration, we also sought to locate the commissioning literature within the evidence on large-scale change in health systems. In particular, we adopted the lens provided by Best et al.’s as a further framework for analysing our empirical findings [4]. These analyses lead to a revised version of Øvretveit’s cycle of commissioning that gives greater emphasis to the development of effective implementation within commissioning processes and to a confirmation of Best et al.’s own acknowledgement that their framework requires continuing development.

### Evidence on the effectiveness of commissioning in enabling change in health and social care

#### Commissioning in the English NHS

Commissioning evolved in the English NHS as an essential element in the adoption of market-style mechanisms in 1991 following the NHS and Community Care Act 1990 [5]. It is an approach rooted in the separation of ‘purchaser’ and ‘provider’ roles, supported by formal contractual mechanisms to regulate relationships between them. This ‘purchaser-provider split’ was, itself, an expression of the wider approach known as ‘new public management’ [6, 7] which sought to introduce competitive forces into the public sector. The underpinning belief of senior NHS policy makers was that purchasing was ‘the engine that would drive the reforms’ through improvements to quality, efficiency and responsiveness [8]. Some 3 years after initial implementation of the purchaser-provider split in the NHS in England in 1991, policy makers expanded the concept and function of ‘purchasing’ and started to adopt the broader role of ‘commissioning’, drawing on the work of Øvretveit [1, 2].

Commissioning encapsulates a series of linked activities described in academic literature as an ongoing cycle [2]. This concept has continued to be used in England to represent commissioning as encompassing the core functions set out in Fig. 1 [9].

Whilst the cycle implies a series of sequential activities, the reality is more complicated, with multiple tasks taking place concurrently and with significant degrees of overlap. Bovaird et al. [10], in a review of models of public sector commissioning, noted the tendency for most approaches to focus on demand-side activities like planning, market shaping and procurement, with limited reference to supporting service delivery and change. Research into NHS health commissioning revealed it to be resource-intensive and ‘laborious’ [11, 12] with inadequate evidence about its respective costs and outcomes [11, 13].

A related critique of health commissioning in the English NHS highlighted its ‘highly relational’ characteristics [14] (such as co-design and stakeholder engagement) and tendency to pay insufficient attention to transactional functions such as procurement, contract review, decommissioning and service redesign [15]. Others have sought to explain the relative weakness of NHS commissioning by its lack of institutional ‘fit’ with health professional and organisational cultures that prioritise collaboration and limit its effectiveness [16, 17].

The English NHS emphasises general practitioner (GP) involvement in commissioning to utilise their knowledge of individual patients’ needs and their gate-keeping role to secondary care [18]. ‘Primary care-led commissioning’ has been a central feature of NHS policy in England since 1991 and its most recent incarnation was the establishment in 2013 of 211 clinical commissioning groups (CCGs), responsible for allocating approximately two thirds of the total NHS budget in England (£73.6 billion in 2017–18) [19]. Research consistently shows that primary care-led-commissioning has been more effective in driving change in general practice and community-based health services than in acute hospital services [20, 21].

The Health and Social Care Act 2012 re-emphasised the role of competition and implicitly encouraged more extensive use of for-profit and third sector providers alongside mainstream NHS-managed services [22]. Yet there has been a parallel emphasis on collaboration and integration, to provide co-ordinated care for the growing numbers of (mainly frail older) people living with multiple long-term conditions [23]. Commissioners have therefore had to explore ways of balancing apparently contradictory pressures: to promote provider competition through contracting and procurement, while simultaneously securing collaborative service delivery through strategic purchasing.

#### Joint commissioning of health and social care

Similar provisions for adult social care accompanied the introduction of an NHS purchaser-provider split in the 1990 Act. However, social care differed in that councils had already been dealing with many external providers in the for-profit and not-for-profit sectors. Indeed, the majority of residential social care was already provided through those sectors, funded predominantly through social security payments [24].

As the interdependencies between the NHS and social care markets began to be acknowledged, the concept of joint commissioning emerged [25, 26]. This was to provide the ‘link between planning and activity’ [24] through formal specification of joint services, engagement of providers, and the shaping of markets to provide a better balance of residential and domiciliary services and reduce the risk of market failure. However, a recent study of joint commissioning over 20 years found there was ‘little evidence to link it to changes in outcomes’ [25]. In this respect, the results of joint commissioning mirrored the limited impact of integration of care provision more generally [10, 27, 28].

There is a great deal of evidence about the wide range of barriers that prevent integrated working being more successful [28]. For example, Waring et al. [29] highlighted the significance of barriers to integrated and inter-professional working stemming from social and cultural differences including those related to knowledge, organisation and power. Weick [30] emphasises the associated role of ‘organisational sense-making’, whereby organisations may use the same terminology but have different interpretations of the issue. Focusing on the role of commissioning, Checkland et al. [31] argue that local sense-making processes will impede top-down solutions unless adequate attention is given to local contexts. Elsewhere, they draw on Scott's [32] analysis of institutions as resting on three pillars (regulatory, normative and cultural-cognitive) to argue that NHS institutions have often focused on the regulatory pillar, without giving sufficient attention to the moral and cultural assumptions that drive behaviour. This has led to a gap between NHS institutions and the process and ambitions of commissioning as a mechanism for delivering change [17].

In summary, NHS and joint commissioning have been understood as a process of service redesign and change that requires a balance between relational and transactional activities. Meanwhile primary care-led commissioning is considered a lever for change in primary care and community health settings, but rarely in others, and professional cultures within and across health and social care constrain efforts to commission integrated care. There has however been relatively little research into the effectiveness or otherwise of commissioning as an approach to bringing about change to whole systems of care, which can encompass multiple CCGs and local authorities, as well as many health and care providers.

### Whole systems integrated Care in North West London

NWL has a population of just over two million people served by eight boroughs (Brent, Ealing, Hammersmith and Fulham, Harrow, Hillingdon, Hounslow, Kensington and Chelsea, and the City of Westminster) and their corresponding CCGs. In 2012, the CCGs and seven boroughs (excluding Hillingdon) established a Whole Systems Integrated Care Programme (WSIC), building on prior local initiatives such as the inner and outer NWL integrated care pilots (ICPs) [33] and Tri-Borough community budgets programme [34]. At the same time, a major reconfiguration of acute hospital services (‘Shaping a Healthier Future’) was planned, together with a further programme to develop primary care as the centrepiece for extending the role of out of hospital services [35].

Soon after WSIC was initiated, the Department of Health announced a call for Integrated Care and Support Pioneers [36]. WSIC became the basis of a bid to this call from 31 partners in NWL sharing a vision ‘to improve the quality of care for individuals, carers and families and to empower and support people to maintain independence and to lead full lives as active participants in their communities’ [37]. This was underpinned by three principles [37]

- People will be empowered to direct their care and support, and to receive the care they need in their homes or local community.
- GPs will be at the centre of organising and coordinating care so that it is accessible and provided in the most appropriate setting.
- Systems will enable and not hinder the provision of integrated care, and ensure that funding flows to where it is needed most.

**Fig. 2:**
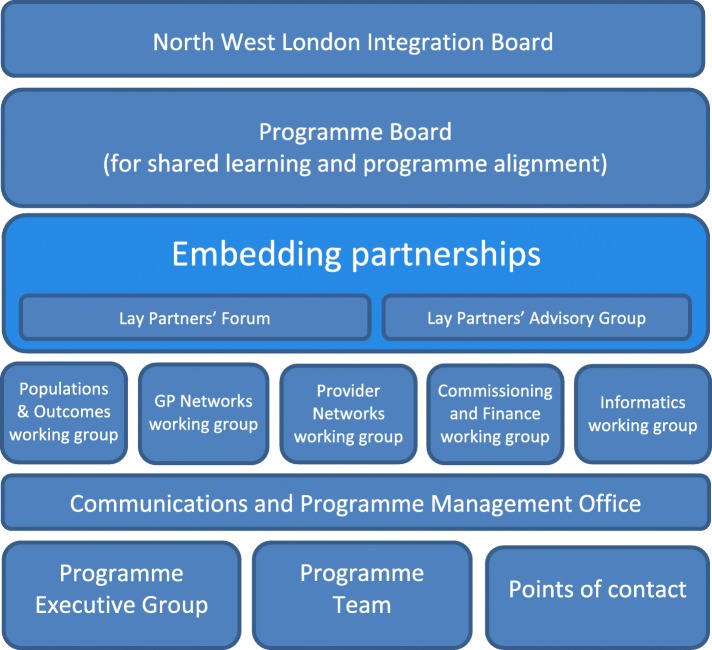
The governance arrangements for the North West London Whole Systems Integrated Care Programme [38]

The original timetable for WSIC specified an eight-month period of ‘co-design to inform local implementation’. Pilot sites, or ‘early adopters’, were to be identified during the last 3 months of this phase, after which they would ‘be ready to commence in shadow form in early January 2014’ and start ‘working together under “whole system” commissioning and provision arrangements to improve outcomes for the local population’ by April 2015 [37]. The governance and implementation structures designed to deliver the WSIC programme were complex and encompassed both the borough and pan-NWL levels [38] (see Fig. 2).

The latter centred on a programme management office and executive staffed by a Strategy and Transformation Team with support from external management consultants and based in the office of the collaborative of the eight CCGs. The CCGs developed a joint financial strategy to support their collective WSIC work, pooling resources allocated to all CCGs by NHS England for ‘transformation’ – £24.9 m was pooled from 2013/14 to 2015/16, of which £7.9 m was spent on management consultancy during the first 2 years [38]. NWL therefore represented a bold and ambitious programme of intended change to local health and care services, with significant reliance placed on the role of commissioning to bring this about.

During the co-design phase, five working groups were established at NWL level to help local areas implement integrated care ‘at scale and pace’ focusing on: populations and outcomes; GP networks; provider networks; commissioning and finance; and informatics. The intention was that preparatory work for whole systems integration would be ‘done once’ across all participating boroughs and CCGs. A cross-cutting ‘Embedding partnerships’ work stream was also established comprising a lay partners’ forum of some 120 people and a lay partners’ advisory group whose members attended each of the five working groups. As well as advising on programme design, lay partners were on all WSIC’s governance bodies and no meeting was considered quorate unless a lay partner was present.

## Methods

### Study design

Our study was commissioned by Imperial College Health Partners and the Collaboration of  NWL CCGs to evaluate the initial design and piloting phases of WSIC from February 2014 to April 2015. The brief [39] stipulated a primarily qualitative evaluation to provide an independent, summative assessment of:

- how the WSIC programme was designed;
- the involvement of local stakeholders in the design process;
- the development and early implementation of early adopter (EA) schemes; and
- the extent to which the WSIC programme appeared to be on track towards its objectives.

A formative element was also required by the funders, to provide ongoing feedback from the research in light of evidence that the momentum of large-scale change often falters and fails as it moves into its implementation phase [39]. The formative component was also an acknowledgement that an independent, academic voice was needed in developing this large-scale, high-profile and costly intervention [40].

The study comprised two phases designed to meet the four research objectives, and to mirror the two phases of development: co-design; and early implementation. The methods employed within each phase are summarised in Table 1. Ethical approval was granted by the research ethics committee of the London School of Hygiene and Tropical Medicine, where the national evaluation of the wider Pioneer programme was located. (LSHTM ethics ref.: 7215).

**Table 1:** Phases of the study and data collected

Phase	Main research tasks	Data collected
*Phase 1 – February to June 2014* *Describing, understanding and assessing the context and processes of WSIC* *(Objectives 1&2)*	Assess co-design of the programme	Research co-design workshop (re overall objectives and approach) 30 × 1-h interviews
	Involvement of stakeholders in co-design	Observation field notes of 60 hrs of meetings or workshops Analysis of programme documentation including WSIC toolkit Review of UK and international literature on integrated care and commissioning Feedback workshop with WSIC stakeholders re initial findings
*Phase 2 – July 2014 to April 2015* *Tracking progress of 9 EA schemes and in-depth case studies of 4 EAs, plus ongoing programme-level monitoring of context* *(Objectives 3&4)*	Development and early implementation of EAs	Research co-design workshop with EA and WSIC stakeholders (re case study approach and selection) 16 × 1 h interviews at pan-NWL level 27 × 1-h interviews in case study EAs Field notes of 60 hrs of meeting observations both in EA case studies and at pan-NWL level Continued analysis of programme documentation On-line survey of members of EA steering committees On-line survey of all GP practices in NWL Feedback workshop with EAs re survey and other data initial findings
	Progress made towards WSIC objectives	Synthesis by research team of all data Comparison of conclusions with WSIC plans Feedback workshop with WSIC core leadership team to share draft report themes and framework Feedback workshop with WSIC stakeholders to share final draft report

### Data collection

Our data collection reflected the WSIC aim of integrating commissioning and care delivery at macro, meso and micro levels [41]. We worked with: commissioners, providers and other stakeholders who were involved as representatives at the pan-NWL (macro) level; leaders and project managers of individual EAs (meso level); and individual providers of care at the user (micro) level. A mixed methods approach was chosen to surface and assess the complexity of WSIC and its wide range of stakeholders [40]. Semi-structured interviews enabled us to explore participants’ perceptions and experiences of the programme’s development. Participants were selected purposively and we conducted 73 one-hour interviews, predominantly face-to-face, with: managers and frontline staff in acute services, CCGs, general practice, community and mental health services, and local authorities; lay partners; and third sector providers (see interview topic guides in additional files 1, 2 and 3). Participation was voluntary and informed prior written consent was obtained. We also observed approximately 120 h of meetings, including the process of selecting EAs, regular WSIC governance meetings, co-design workshops, and events held to refine WSIC plans. We undertook documentary analysis of programme materials and conducted a rapid review of UK and international literature on integrated care and commissioning. Finally, as part of the formative approach, the team conducted co-design workshops with a cross-section of stakeholders from the NWL health and care system.

Four EAs were selected as case studies [42] on the basis of geography, contextual differences, target population characteristics, partners delivering the intervention, readiness for implementation, scale of the initiative, and the extent of social care involvement [38]. To complement the case studies, we conducted two on-line surveys. The first covered all steering committee members for each of the nine EAs (November to December 2014). It explored perceptions of progress, the value of the initial co-design process and outcomes, and the contribution of different stakeholder groups. A 60% response rate was obtained. A second survey – of GP practices – was conducted to explore GPs’ awareness of WSIC, as well as their views of its plans and local requirements to realise them. A covering letter with a hyperlink to the survey was distributed by CCGs from March to May 2015 and secured a response rate of 39%.

### Data analysis

All interviews were recorded, transcribed and then coded using NVivo 11. Blind coding was undertaken by the research team to check consistency. A framework analysis approach [43] was applied to the coding, using high-level themes to categorise the data. Themes were first created deductively based on the findings of the literature review and subsequently refined inductively based on a pilot coding and analysis of a randomly selected subset of 15 interviews. The final themes used in the framework analysis included: transactional and relational functions of health commissioning drawn from Øvretveit’s cycle; professional and cultural aspects of WSIC development as deduced from our review of literature on health and joint commissioning (including the role of GPs and primary care, and involvement of local government); key organisational components of integrated care (including finance, governance, model of care); historical and contextual factors; and views about the future of WSIC. A draft narrative account of EA ambitions and development was prepared for, and checked with, each of the four case study sites. Data were analysed by the research team in four internal workshop sessions, exploring findings in relation to the three WSIC principles described above. Emerging conclusions were tested with WSIC stakeholders in two additional workshop sessions as part of our formative approach, prior to completing the research report.

### Limitations of the research methods

The scale of the WSIC programme presented challenges to obtaining detailed understandings of activities across the entire initiative. Despite conducting a large number of interviews, we were unable to engage with front-line staff to the extent we had anticipated, largely because of delays in establishing integrated care services on the ground. The team was aware that the formative component of the study contained a number of risks including those of identifying too closely with the success of WSIC and being pressed for definitive findings before our evidence had been fully analysed. Consequently, we were careful to restate the summative as well as formative objectives of the research in feedback to WSIC and to highlight the degree of confidence we placed on different findings as they emerged.

## Results

### Strategic role of commissioning

The WSIC programme was ambitious in its vision and objectives, with five domains of intended outcomes ultimately identified: quality of life; quality of care; financial sustainability; professional experience; and operational performance [44]. However, performance measures set by NWL commissioners for WSIC emphasised national targets: reducing avoidable emergency admissions to hospital; and reducing emergency department attendances – by 10 to 15% and up to 30%, respectively [44]. The difficulty WSIC commissioners experienced in setting measures that encapsulated their broader aspirations reflected their more general struggle to articulate exactly what they were trying to do and by when: *‘There’s just so much change to implement all at once, that their target date of having things go live on the first of April next year is, I think, completely unrealistic’* (I1). This reflected concern emerging from our data about the criteria used for determining how far programme intentions were being realised. We concluded such concerns substantially reflected the very high-level nature of WSIC aims and ambitions.

The programme described itself as ‘commissioner-led’, although which set of commissioners was actually leading appeared to be contested. For some, WSIC was deemed to be led by GPs in their role as local commissioners: *‘The Whole Systems is GP-led. But it’s led by GPs as CCGs … those GPs that have a strategic view’* (I2). Others cited the overall CCG organisations as being in the lead, given their statutory NHS planning function: *‘we are, as commissioners, also having to set out a strategy for the system’* (I3). Yet others saw ambiguity and conflicts of interest in CCGs, as GP-membership organisations, undertaking the leadership role: *‘your CCG colleagues are both building the new commissioning approaches, but also have got one eye on how they trade in the provider network and that is really causing a lot of tensions’* (I4).

During our research, governance and strategy at the NWL level were driven primarily by the Collaboration of CCGs, its programme executive group and programme team (see Fig. 2). There was less evidence of input from local government and other partners. *Although it was a very co-designed programme it’s probably fair to say in the early days right through to autumn last year [2014] they would probably have said it was a clinical commissioning group, commissioner-driven conversation, even though they were full partners* (I5).

In our survey of EA steering groups in late 2014 (Table 2), 87% of respondents reported that CCGs had been ‘very’ or ‘extremely’ involved in designing their local EA, and 83% for the WSIC programme team, 31% for NHS front line staff and 58% for adult social care commissioners (Table 3).

**Table 2:** Results from early adopter survey: ‘How involved have local commissioners been in the design of your early adopter project (from February 2014 to now)?’

	Local authority adult social care	Local authority public health	CCG	NHS England (London)
Extremely involved	14%	5%	57%	3%
Very involved	44%	25%	30%	12%
Moderately involved	24%	3%	9%	23%
Slightly involved	15%	39%	2%	32%
Not at all involved	4%	27%	1%	30%
*Total*	*100%*	*100%*	*100%*	*100%*
*N*	*80*	*59*	*82*	*77*

Source: Survey of WSIC EAs conducted from November–December 2014

**Table 3:** Results from early adopter survey: ‘How involved have these other groups been in the design of your early adopter project (from February 2014 to now)?’

	Patients, service users, carers	Lay partners	Voluntary sector representatives	NHS front line staff	WSIC programme team
Extremely involved	35%	39%	25%	9%	51%
Very involved	31%	39%	48%	22%	32%
Moderately involved	26%	14%	20%	48%	16%
Slightly involved	7%	8%	7%	20%	1%
Not at all involved	1%	1%	0%	1%	0%
*Total*	*100%*	*100%*	*100%*	*100%*	*100%*
*N*	*81*	*80*	*81*	*81*	*81*

Source: Survey of WSIC EAs conducted November–December 2014

By the end of our study in April 2015, and despite major investments of time and money in designing the programme, WSIC was over a year behind schedule and only two of nine EA schemes were even partially under way [38]. WSIC’s bold and proactive vision appeared elusive in implementation.

### Relational and transactional elements of commissioning

The scale and complexity of the NWL system brought with it high transaction costs. The programme executive alone held 25 meetings between October 2013 and May 2015, an indication of the time commitment expected in one element of the commissioning cycle: planning. As one respondent noted: *“[it is] costing us a fortune in terms of man and woman hours, a shocking amount of time it is getting to nearly ridiculous proportions”* (I6). At the same time, WSIC leaders emphasised that they had sought to expedite matters by adopting an approach that transcended usual commissioning arrangements: ‘*we completely disregarded the commissioner–provider split in doing it [developing WSIC], and that’s caused some angst from some of the local authorities and from some of the CCGs, saying, “You’re running completely roughshod over all our commissioning responsibilities”. And I’m glad we did because, if we hadn’t, we would have massively constrained what people came up with’* (I7).

The co-design phase of WSIC was intended as a mechanism for organisational development, to develop integrated care ‘tools’ and to foster local ownership: *‘[it] came about because we were clear that this was only going to work if it was a journey that everyone came on, but we also had to strike the balance between doing some of the design thinking once that local areas could then hang their own initiatives off’* (I8). However, this was experienced by some as burdensome: *‘we’ve almost been too engaged in the sense that there’s been so many meetings and so much activity that one is invited to which we can’t always service’* (I9). For others, co-design work was an expensive process (reflecting the cost of support commissioned from external consultants) and its value for money was questioned: *‘This entire programme, we spent probably more than £2m on [name of company] along with the toolkit … I question the balance, and management consultancy – by definition they come and go’* (I10).

The extent of stakeholder involvement in WSIC design work was demonstrated by responses to our survey of EA steering group members (see Tables 2 and 3). WSIC explicitly embraced an inclusive approach to build support for new models of care, but it was heavily front-loaded into the co-design process. We reported back to WSIC leaders the risk of stakeholder involvement tailing off in spring 2015 as implementation began and resources for supporting implementation were spread across nine EAs. The significant attention paid to relational work within WSIC was particularly evident within the Embedding Partnerships work stream: 78% of steering committee respondents reported lay partners had been ‘very’ or ‘extremely’ involved in local project design (Table 3). The continuing commitment and resource allocated to this work stood out compared with other Pioneers [28].

The early development of EAs continued to be largely relational in nature – for example in designing local models of integrated care – with much less emphasis on transactional issues such as negotiating needs-based budgets, rewriting contracts to procure new models of care, designing and implementing new workforce roles, or piloting multidisciplinary team working.

Transactional elements of implementation were not wholly neglected, being addressed in the phase one, co-design work streams and captured in a ‘toolkit’ for integration [44] which received significant input from external consultants. This toolkit included resources to support: contracting and procurement, the calculation of population-based budgets, and approaches to collecting and using data for planning and delivering integrated care. Consistent with the objective of ‘doing the design work once’, 75% of steering group respondents thought the toolkit had been ‘very’ or ‘somewhat’ helpful when designing their EA.

We concluded that WSIC was highly relational in its approach, including in the way in which it sought to design and develop transactional elements of the programme. It seemed as though the anatomy of integrated care was constantly attended to, yet the physiology – the actual life-blood and practical functioning of the desired new approach to care – remained elusive or perhaps just too difficult to enact.

### Role of GPs and primary care in commissioning

Although ostensibly CCG and local authority-owned and led, the WSIC programme was in reality designed and managed from the programme’s NHS headquarters. Even when primary care contributions were identified, only a minority of GP leaders were seen to be fully engaged. Whilst 72% of GP survey respondents had heard of WSIC (*n* = 93), only 25% of them considered themselves to be ‘quite’ or ‘very’ involved in it (*n* = 23). Workload pressures were considered by GPs to be the main impediment to WSIC progress, suggesting a lack of engagement by the programme of frontline GPs or responsiveness to their local perspectives and concerns. Thus the extent to which WSIC had become part of the physiology of local health systems through the mechanisms of primary care-led commissioning was limited. Respondents within primary care argued for their perspective as providers to be heeded within WSIC: *‘we’re a membership organisation of general practices … so in part I have a role to engage with and bring along all of general practice in the work we are doing’* (I3). This was acknowledged by the central strategy and transformation team: *‘it has to be owned by them [local areas], otherwise it will not work, really; it will just fall over as they get into the next phase’* (I2). This view also seemed prescient in anticipating the struggle that WSIC would have in moving from pan-NWL planning and design to the local implementation of integrated care.

The central role of the GP in coordinating care for people was one of WSIC’s three core aims, a feature some criticised as an overly medical focus for a ‘whole systems’ initiative: *‘The programme is being led by CCGs, albeit with support and sponsorship from local authorities. To the outside world, it can appear health-led which can cause issues’* (I3) One work stream focused on developing networks of GP providers consistent with national objectives of ‘scaling up’ general practice and community services to support the shift of services from hospital settings [45, 46]. Even here however, it seemed that the drive came more from the collaboration of CCGs and central WSIC team, than from general practice itself: *‘We’re still in exactly the same phase that we would be, which is the commissioners write the spec, they decide they’re going to procure, and then we all bid for that, like any other system [ …*.] *Similarly, the commissioners are designing when and how the providers should be engaged – that is not being provider-led’* (I11).

Despite extensive lay partner and other stakeholder engagement in its design and development phase, our overriding perception of WSIC was of a large NHS strategic management project, not a primary care-led approach with significant health and social care provider engagement. In practice, much of the impetus, decision-making, and priority-setting came from the senior management of the collaboration of CCGs more than the wider body of the local health and care system.

### Commissioning and culture in health and social care

WSIC encountered a series of cultural clashes. First, there was the conceptualisation of WSIC as a ‘strategic management project’, lent weight by leadership from WSIC ‘headquarters’, significant investment in external management consultancy support, and the consequent use of a highly programmatic approach to project planning, management and reporting. The associated use of temporary project managers at the level of the individual EAs was considered by some to be problematic, albeit we observed that their skills were important to keep the programme development moving: *‘It’s crazy, yes, because they’re temporary, and then when they go away there’s nobody there that has the institutional knowledge or the ability to carry it beyond … you have dedicated people, but you just don’t have enough resources and leadership and the ambitions are too high for what they want to achieve by when’* (I12). The senior managers leading WSIC were influential and respected but both of the two principal management architects had moved on before our study concluded.

This ‘project-ness’ [47] was frequently noted by our respondents as being an uncomfortable feature and even alien to the culture of health and social care professionals in NWL, particularly in relation to the use of management consultants. We were told, for example, that: ‘*[Name of company], having got the brief, are very clear about what they think is the answer particularly around ACPs [accountable care partnerships] and capitated budgets, whereas others increasingly have doubts about that’* (I13) although there was acknowledgement of the rigour and attention to process that this involvement brought: ‘*I feel part of something quite strong and I think that’s the level of capacity that’s invested. I always wonder how it’s invested and where the money comes from but I think the people generally who lead it, I think are for the most part very strong and independent arbiters really, which I think is a very strong role that they play’* (I14).

The cultural dissonance between primary care-led commissioning, and the extensive use of programme management and external consultants, was summed up by a hospital clinician: ‘*I think it should be clinician driven, clinician led, clinician and patient driven. Why do we need to pay an expensive external agency to do what is basically motherhood and apple pie?’* (I15).

Second, there were cultural differences between the NHS and local government. As explored elsewhere [38], WSIC was presented as a joint undertaking across the whole NHS and local government system with a focus on wellbeing, prevention and independent living. In reality however, it focussed on projects intended to address short to medium term NHS concerns, namely avoiding admissions to hospital or reducing time spent in hospital. This was described as *‘they’re [social care managers] in the room but they’re not at the table’* (I16) and *‘I was astounded at … the lack of capacity in local authority feeding into the whole systems, at the low level of relationship building, common purpose, vision and strategy, rather than focusing on the technical aspects of WSIC, which the external consultants are driving’* (I4). We concluded that explanations included: pressures on local government finances that prevented senior managers attending the multiple workshops and meetings integral to the WSIC approach; its strong NHS management focus; and the failure to engage local political leaders.

Third, WSIC’s commissioner-led approach meant that local providers of health and social care felt that they were not always sufficiently involved in the programme’s planning work: *‘One of our problems in [borough] has very much been that this is not being led by the providers and the providers have been passive recipients … it’s us as commissioners really pushing them’* (I17). This may reflect providers’ belief that change was unlikely to happen, or that they were waiting for evidence of serious commissioner intent to invest in change. *‘Designing the care planning processes has not particularly involved providers, not to the extent that they feel ownership of it [ …*] *I think they’ve set themselves up for something that they’re not able to deliver’* (I18). Thus WSIC’s overly relational approach to commissioning led to provider engagement that was significant in one sense (senior managers and clinicians were often at key meetings) but was not rooted back into the operation of individual health and social care organisations, and hence unlikely to lead to practical changes to services at local level.

## Discussion

### Commissioning integrated care in north West London

The WSIC programme was led by commissioners with NWL-wide responsibilities, who were heavily dependent on CCGs and local authorities to use strategic purchasing capabilities to achieve better integrated care. Yet, by May 2015, the programme was falling significantly behind its own delivery timeline. Despite WSIC’s bold vision, respondents to our evaluation struggled to describe precisely what changes to day-to-day provision of services were intended and when they would be achieved.

NWL commissioners relied heavily on relational aspects of commissioning as the main route for change. Even where attention was given to transactional work (e.g. exploring how to set population budgets, design contracts, determine information governance), this was approached through highly relational means (such as co-design workshops), which stakeholders struggled to translate into operational practice. WSIC leaders assumed that engaging stakeholders in NWL-wide co-design work streams would result in the latter becoming ambassadors for change in local EAs, but this expectation was neither explicitly articulated nor supported.

WSIC leaders likewise seemingly assumed that senior GP commissioner involvement in WSIC governance and planning would permeate the wider general practice community, but our survey of all GP practices in NWL suggested otherwise. WSIC sought to establish GP provider networks across all eight CCG areas, but this flew in the face of evidence on the need for such organisations to be professionally developed from the bottom up [18, 48].

The extensive use of external consultants was a key factor in some of the cultural clashes identified above and the scepticism among some NHS and social care professionals about the value of this investment. A further limiting, cultural factor was WSIC’s NHS-constructed view of care integration and failure to embrace a more fully whole systems approach. In our fieldwork, we found little reference to joint commissioning within the WSIC framework, although there were examples of ongoing initiatives at the borough level. Whilst it might be argued that WSIC was an NWL-wide exercise in joint commissioning, local authority involvement was most limited at that level. Local authorities lacked capacity to engage fully with WSIC, and did not consistently identify themselves as ‘North West London’ (having eight independent boroughs and two local government alliances of councils in the area) in the same way as the NHS, which was working to its prior regional footprint.

The NHS leadership within WSIC set unrealistic objectives and timescales for major change – seemingly beguiled by the latest policy fashion [49] – and failed to join up the interdependent strategies of WSIC and ‘Shaping a Healthier Future’ [35] into publicly narrated and detailed programmes of complementary change. Equally, it remained to be seen whether borough-level joint commissioning would kick in as the focus of WSIC moved to rolling out EAs across local areas. The evidence from both the initial (NWL) stage of work and the literature on joint commissioning suggested this would be a significant challenge.

The weaknesses of health commissioning identified in our review of the literature were found to be present in NWL’s enacting of its WSIC programme, and contributed collectively to the significant delay in implementing the vision for integrated care locally. WSIC was also an attempt to achieve large-scale organisational change. Best et al. [4] suggested a set of ‘simple rules’ for undertaking large-scale change i) engage individuals at all levels in leading the change efforts; ii) establish feedback loops; iii) attend to history; iv) engage physicians; and v) involve patients and families.

Applying these rules in NWL, it can be asserted that:

- significant effort went into engaging some people at some levels in plans for change;
- the project planning approach was extensive and included apparently rigorous ‘checkpoints’ and monitoring;
- WSIC built on prior integrated care and community budgeting initiatives locally, as well as participating in national pilot schemes;
- the WSIC programme was located within a policy of primary care-led commissioning committed to strong general practice involvement; and
- there was a major commitment to recruiting, training and working with lay partners.

Less evident however were:

- successful engagement of front-line health and social care staff, their managers and union representatives in detailed planning for new ways of providing and staffing services;
- involvement of local politicians in WSIC planning and governance;
- setting graduated and realistic outcome measures;
- learning sufficiently from prior local experience of pilots that had demonstrated the time required to change service delivery patterns across multiple professions and the limited prospects of affecting emergency admission rates;
- engaging the majority of clinicians employed in community and hospital settings in the implementation of EAs; and
- involving the public, patients and carers in actual implementation of service change, as opposed to having intensive but narrow engagement in programme planning and governance.

At the same time, however, some of our findings could not be accommodated within Best et al.’s five rules [4]. This was not unexpected. The authors, themselves were explicit about the limitations of their paper as, first, a small-scale rapid review commissioned by Saskatchewan policymakers; while also emphasising that a ‘second, and perhaps more important, limitation of this study was not with the methodology but with what was not in the literature or identified by our consultation group. The five simple rules described here may be *necessary* for large-system transformation but are probably not *sufficient*’ (p445). We would concur with this conclusion. While we have a more limited focus here which, together with space limitations, does not allow for a comprehensive re-assessment of the ‘five rules’, it is nonetheless clear that some critical dimensions of the NWL experience are not captured by them or the literature review from which they were derived. For example, the rules give very little recognition to the importance of cultural, behavioural and cognitive factors which can both facilitate and impede commissioning processes and the implementation of large-scale change in health and related services [30–32].

### The potential and limitations of commissioning in delivering large-scale change and integration

In a publicly funded health system, it is vital that there is a body that sets priorities, plans how best to use its resources to meet identified needs, puts these intentions into action, and accounts publicly for its performance [16]. The English NHS continues to use new public management’s purchasing and contracting functions for this purpose, working within Øvretveit’s commissioning cycle. Our study highlights the consequences of giving insufficient attention to implementation in that cycle and especially the need for commissioners to enable, support and performance manage the delivery of procured services, while working closely with providers at all times.

The critical first stage of implementation is to reach consensus across commissioners, providers and service users about ‘what is it we intend to do’ so that the exact nature of the service change can be more precisely articulated, planned and enacted for staff, users and carers. Commissioning aims in WSIC were too often poorly specified, and then subject to overly optimistic and blunt measures of progress – such as avoided emergency admission to hospital, or roll out of EAs in 6 months. As a result, such aims are inevitably doomed not to be achieved [50]. The work of commissioners in convening the different actors (e.g. funders, regulators, providers, service users) to review, plan and (re) design approaches to care forms part of the service design stage of the commissioning cycle, and needs to link closely with subsequent implementation work as part of the process of ‘making it happen’. This focus on providers and commissioners working together to clarify commissioning goals and outcomes, including their implications for provider roles and behaviours, and the implementation of new services, requires acceptance that the ‘purchaser-provider split’ is neither neat nor clear, but an intellectual distinction across which significant discussion, collaboration and joint work are required as plans are developed and implemented.

## Conclusion

We concluded that the highly relational co-design phase in NWL could take the commissioning process only so far, and that commissioners have an equally important role in piloting and implementing new services to ensure the presence of the necessary support, resources and management expertise. Explicitly identifying implementation processes and responsibilities within the commissioning cycle emphasises the vital need to engage service providers from the outset (including front-line staff most affected by service changes). As our research was concluding in April 2015, the WSIC programme was setting up a ‘change academy’ to support implementation of the EAs, a highly appropriate approach, but perhaps somewhat late in the overall change process.

Richard Bohmer [51] has reviewed international experience of attempts to transform health services and he echoes our findings in NWL, highlighting the limitations of commissioning in market-based approaches: ‘experience shows that although a changed market may be a helpful precondition to local performance improvement, it hardly guarantees effective operational change.’ Bohmer asserts the importance of sustained attention to multiple marginal changes required for the implementation of ‘transformed’ services, over a long period of time, guided, resourced and supported by senior leaders who can also ensure the commitment of middle managers and multidisciplinary teams. His argument reinforces the similar conclusions of Dixon-Woods et al. [47] and this need to secure large-scale change through incremental steps was also highlighted in international consultancy advice to NWL as our study ended.

**Fig. 3:**
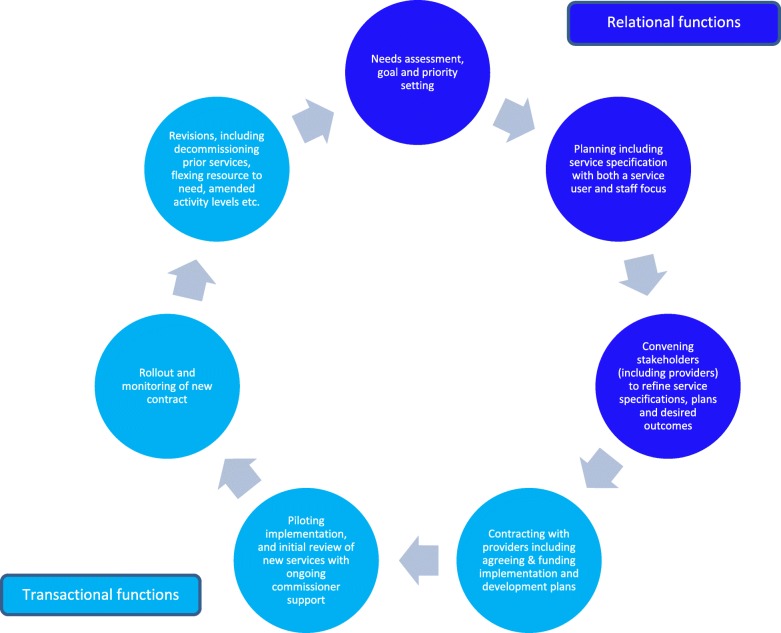
Proposed revised commissioning cycle

We propose therefore a revised cycle of commissioning (see Fig. 3) that retains its focus on assessing needs, planning, engaging in service (re) design, procuring (and decommissioning) services. It also builds on work by Oxford Brookes University [52] that incorporated Deming’s [53] plan-do-review-act approach into the cycle of commissioning and includes change and budget management functions. Similarly, our approach reflects Murray’s [54] suggestion that a ‘purchasing cycle’ should operate within the commissioning cycle, to provide a more explicit focus on the ‘delivery’ function. Our proposed commissioning cycle emphasises the responsibility of commissioners to:

- define specifically what changes to services are intended;
- convene stakeholders to plan for and support implementation continuously; and
- see outcomes as something for which commissioners and providers are jointly accountable.

This model shares much with the ‘accountable care’ approach currently promoted as the solution to the vexed business of NHS commissioning [19, 55] and for the purchasing of publicly funded health care in the US [56].

Securing significant local change to health and social care services (either separately or together) will always be difficult, and require sustained and detailed attention on many fronts. Competent commissioning may help ensure appropriate monitoring and review of current services, the design and planning of necessary changes, and setting of priorities for funding. It has its limits however, and these need to be acknowledged, especially in change at the ‘scale and pace’ so often exhorted by national leaders. In addition, healthcare commissioning must shift the balance of its attention from the relational towards the transactional, and work constantly with service providers as well as users to specify, agree, support, coordinate and see through the implementation of change. Without this, researchers will continue to produce critical verdicts on the progress of large-scale and costly attempts to develop better integrated care. Knapp and Wistow [57] conceptualised commissioning as providing the missing link between planning and activity. Both our study and the literature emphasise that the need to support implementation is critical and continues to be insufficiently recognised or developed. This need remains the weakest link in commissioning and thus in health systems’ ability to secure transformative change in local health and care services.

## Additional files

Additional file 1: Interview guide used for leaders in WSIC evaluation (DOCX 19 kb)

Additional file 2: Interview guide used for senior stakeholders in WSIC evaluation (DOCX 14 kb)

Additional file 3: Interview guide used for early adopter interviews in WSIC evaluation (DOCX 14 kb)

## Abbreviations

ACP: Accountable care partnership
CCG: Clinical commissioning group
EA: Early adopter
GP: General practitioner
ICP: Integrated care pilot
LSHTM: London School of Hygiene and Tropical Medicine
NWL: North West London
PCT: Primary care trust
UK: United Kingdom
WSIC: Whole systems integrated care
