# Supplementary material for: Evaluating the design and implementation of the whole systems integrated care programme in North West London: why commissioning proved (again) to be the weakest link
Source: BMC Health Serv Res. 2019 Apr 15;19:228. doi: 10.1186/s12913-019-4013-5 (PMC6466786; doi:10.1186/s12913-019-4013-5)
Supplement: Supplementary file 1 — Interview guide used for leaders in WSIC evaluation (DOCX 19 kb) [file 12913_2019_4013_MOESM1_ESM.docx]

**NW London Whole Systems leaders interview schedule**

**Introduction**

- Thanks, confidentiality, etc.
- Ask if interviewee has received the information sheet and confidentiality form, and provide them with new copies if necessary.
- What we are focusing on in our evaluation (topic areas), our methodology/general approach, rough idea on when we expect outputs.

**The interviewee**

- What is your current role in the Whole Systems pioneer?
- What was your involvement in the Inner North West London pilot? Were you involved in any way with the Outer North West London pilot?
- How does that role compare with your involvement in the Whole Systems pioneer?

**The Whole Systems programme**

- How would you describe the Whole Systems programme to us in a nutshell? (PROMPT: What is the programme’s underlying approach to integration, or model of integration?)
- How does the Whole Systems programme differ from the Inner and Outer NW London ICP(s)?
- What lessons have been learnt from the Inner and Outer NWL pilots? Have they all been incorporated into the Whole Systems programme in ways that are consistent with your understanding of that learning?

*OPTIONAL:*

- - What was the most successful part of these earlier pilots? What has the pathway been from these successful elements to the Whole Systems?
  - Aside from you, which other leaders from the INWL pilot are involved in WSIP? What role do they play?
- What has been the relevance of the community budgets pilot to WSIP?
- What factors were important in shaping the Whole Systems programme and enabling it to be submitted as a pioneer bid?  What did and do you expect pioneer status to add?
- What will success look like for the pioneer? What criteria would you suggest adopting for the first couple of years and in the longer term (say, 5 to10 years’ time)?
- Is there a plan B?  (PROMPT: What happens if out-of-hospital services are expanded, but activity levels of unplanned admissions remain high?)
- Is any new or innovative governance of purchaser and provider functions being developed as part of the Whole Systems pioneer? What is different from the INWL pilot?
- Is there anything unique about NW London that has made setting up the pilot easier or harder in terms of governance – for example, how far are the tri-borough arrangements a helpful feature of the local governance of integration?
- How are you involving user (and carer) views and is this different from before?

**The co-design phase and development of early adopter expressions of interest (EoIs)**

- Were you involved in the co-design phase in any way?
- What do you think of the toolkit, both in terms of the process of creating it and in terms of the final product
- Have you found the toolkit to be useful? What criteria would you use to judge its success? Would other have different criteria?
- Do you know if the early adopters and other providers are finding it to be useful in practice?
- From your perspective, how is the early adopter initiative developing? Does the preparation of EOIs have sufficient developmental and project management support?

**Involvement of providers**

- How willing have different providers been to be involved in the pioneer?
- How involved has the voluntary sector been? Has housing been involved, either at a strategic level or in the preparation of the early adopters’ EOIs?
- What is the balance of incentives and disincentives for different categories of provider for engaging in WSIP?
- How is the Whole Systems team ensuring that aspirations at a high level are relevant to the day-to-day cultures and behaviours of staff in direct contact with patients/service users? How will the programme help promote multidisciplinary working at that level?
- What has been your experience managing the frequently perceived tension between integration and national competition policy?
- Where does the integration of public health take place in the Whole Systems programme?

**Workforce**

- Does the Whole Systems pioneer involve any workforce innovations?

**Information systems**

- Are local information systems fit for purpose in terms of supporting integrated care and governance?
- Can you foresee being able to share information across organisations and services about individuals to support personalised and coordinated care?
- Is the IT system that was developed as part of the Inner North West London ICP still in use? If not, what led to it being abandoned?

**Financial arrangements**

- What, if any, new financial arrangements are to be put in place? (PROMPT: pooled budgets, capitation, incentives for performance, etc.)
- Are any currently in operation?
- How will risk be allocated between commissioners and providers with the capitated budget?

**Better Care Fund**

- How important is the BCF likely to be in helping to achieve this Pioneer’s objectives? Are you adding other resources to the BCF pool?
- Do you feel that the national performance criteria for the BCF are consistent with the objectives of the Pioneer?

**Progress**

- Overall, how are things going so far in terms of developing and implementing the Pioneer as a whole? Are you on track?
- How can any obstacles be removed or mitigated by the pioneer? Are their areas where support from central agencies will be necessary?
- Is there anything you would like to add on how you have found Pioneer status so far?

*OPTIONAL:*

- - Are there any particular problem areas in general or in relation to the BCF progress measures?

**Looking forward**

- How will you define success of the programme as a whole?
- How will you define the success the early adopter phase? Can an early adopter fail?
- How does the Whole Systems programme take account of the fact that different providers, such as mental health or community services, will have different starting points compared, for example, to an acute trust, which may already have been embarking on its own integration activities? (PROMPT: ACOs)

*OPTIONAL:*

- - If the project is a success, what will it look like in 12 months’ time?

- Are there any other comments you would like to make on the issues we have covered today? Have we missed any significant topics in our questions?
