# Supplementary material for: Evaluating the design and implementation of the whole systems integrated care programme in North West London: why commissioning proved (again) to be the weakest link
Source: BMC Health Serv Res. 2019 Apr 15;19:228. doi: 10.1186/s12913-019-4013-5 (PMC6466786; doi:10.1186/s12913-019-4013-5)
Supplement: Supplementary file 2 — Interview guide used for senior stakeholders in WSIC evaluation (DOCX 14 kb) [file 12913_2019_4013_MOESM2_ESM.docx]

WSIC senior stakeholder interview

Phase II (Sep-Oct 2014)

- How would you describe the Whole Systems programme at the present time?
- In what ways have things moved on since we last spoke in the spring?
- What has cheered you about progress over that time?
- What has concerned or frustrated you?
- What do you see as the main challenges facing the Whole Systems programme at present?
- Have you received the support you anticipated from the national partners in the pioneer programme?
- What has been the impact of the national policy environment (e.g. cost pressures in various parts of whole system, Better Care Fund, electoral cycle, other national policies)?
- What has been the impact of the local policy environment (e.g. acute hospital reconfiguration; other out-of-hospital initiatives)?
- How is buy-in at the moment to the Whole Systems programme by the various stakeholders, e.g., acute trusts? Thinking about some key ingredients of the programme: Local government willingness to sign up to pooling budgets? GP readiness to form networks? (Mention survey.)
- What are the particular challenges for the central team?
- What is your take on the readiness (or not) of the early adopters?
- Thinking about the timetable for establishing integrated care as a mainstream way of working on the ground, there have been some changes in terms of when business plans are due, etc. Are there any lessons that would be suggested by this, both for the Whole Systems programme and for non-pioneers?
- Is there anything else you would like to make the evaluation team aware of?
