# Supplementary material for: Evaluating the design and implementation of the whole systems integrated care programme in North West London: why commissioning proved (again) to be the weakest link
Source: BMC Health Serv Res. 2019 Apr 15;19:228. doi: 10.1186/s12913-019-4013-5 (PMC6466786; doi:10.1186/s12913-019-4013-5)
Supplement: Supplementary file 3 — Interview guide used for early adopter interviews in WSIC evaluation (DOCX 14 kb) [file 12913_2019_4013_MOESM3_ESM.docx]

**Early Adopter (EA) Interviews October/November 2014**

**Interview Schedule**

1. How would you describe the aims of the EA?
2. What is the model of care on which it is based?
   1. How and by whom was the model of care developed?
   2. How far can the model of care be described as evidence-based?
3. What are the governance arrangements for the EA?
   1. Who is accountable for its design and delivery and to whom?
   2. Through what structures/processes is that accountability expressed?
   3. Does the Health and Wellbeing Board have a role in holding the EA to account? If so, how far is this role exercised proactively?
4. What infrastructure is available from the Whole Systems Integrated Care (WSIC) programme team and/or local sources to support the development and implementation of the EA? Is it sufficient in quantity and quality?
5. How essential is the support of the WSIC programme team to continuing progress in implementing the EA?
6. As the whole systems integration initiative has moved into the early adopter phase, how far and in what ways has the balance of influence and input changed between
   1. the central and borough level
   2. NHS, local authority and lay (user) interests
7. What are the main issues that have to be addressed and finalised before the EA becomes operational? What is the EA’s level of readiness at the present time in relation to
   1. IT and information sharing (be sure to distinguish between availability of historical activity data and systems for real time sharing of patient information for care planning, etc.)
   2. Formation of GP networks
   3. Formation of provider networks
   4. Capitated budgets
   5. Joint commissioning
   6. Culture change and joint working at the level of service delivery
   7. Provider organisation engagement in and ownership of the EA objectives and model of care
8. When do you expect the EA to begin to deliver changed patterns of work and user experience? How will you know?
9. What are the principal factors that are enabling you to meet the challenges of developing and implementing the EA? How confident are you of success? Over what timescale?
10. NWL is part of the national Integration Pioneer programme.
    1. What has Pioneer status meant to you so far?
    2. In what ways, if any, has it facilitated the EA’s progress?
    3. Is there anything central government should do to remove barriers, support enablers or otherwise contribute to the implementation of your EA and/or the WSIC programme more broadly?
